# Hand Hygiene Compliance at Two Tertiary Hospitals in Freetown, Sierra Leone, in 2021: A Cross-Sectional Study

**DOI:** 10.3390/ijerph19052978

**Published:** 2022-03-03

**Authors:** Gladys Nanilla Kamara, Stephen Sevalie, Bailah Molleh, Zikan Koroma, Christiana Kallon, Anna Maruta, Ibrahim Franklyn Kamara, Joseph Sam Kanu, Julian S. O. Campbell, Hemant Deepak Shewade, Saskia van Henten, Anthony D. Harries

**Affiliations:** 1 Joint Medical Unit, Ministry of Defence, Republic of Sierra Leone Armed Forces, Freetown 00232, Sierra Leone; stevesyllo@gmail.com; 2 Department of Clinical Research, Sustainable Health Systems, 34 Military Hospital Research Center, Freetown 00232, Sierra Leone; bmollehshs@gmail.com; 3 Department of Laboratory, Diagnostics and Blood Services, Ministry of Health and Sanitation, Freetown 00232, Sierra Leone; zikankoroma@gmail.com; 4 Infection Prevention and Control Unit, Ministry of Health and Sanitation, Freetown 00232, Sierra Leone; christy.conteh@yahoo.com; 5 Infection Prevention and Control, World Health Organization Country Office, Freetown 00232, Sierra Leone; marutaa@who.int (A.M.); ikamara@who.int (I.F.K.); 6 Directorate of Health Security and Emergencies, Ministry of Health and Sanitation, Freetown 00232, Sierra Leone; samjokanu@yahoo.com; 7 Ola During Children’s Hospital (ODCH) and Princess Christian Maternity Hospital (PCMH) Laboratory, Ministry of Health and Sanitation, Freetown 00232, Sierra Leone; juliansocampbell@yahoo.com; 8 Division of Health System Research, ICMR-National Institute of Epidemiology (ICMR-NIE), Chennai 600077, India; hemantjipmer@gmail.com; 9 Department of Clinical Sciences, Institute of Tropical Medicine, 2000 Antwerp, Belgium; svanhenten@itg.be; 10 Centre for Operational Research, International Union Against Tuberculosis and Lung Disease (The Union), 75001 Paris, France; adharries@theunion.org; 11 Department of Clinical Research, Faculty of Infectious and Tropical Diseases, London School of Hygiene and Tropical Medicine, London WC1E7HT, UK

**Keywords:** hand hygiene compliance, hand hygiene opportunities, Sierra Leone, alcohol-based hand rub, WHO hand hygiene standard observation tool, SORT IT, infection prevention control, hospital-acquired infections, operational research, AMR

## Abstract

Hand hygiene actions are essential to reduce healthcare-associated infections and the development of antimicrobial resistance. In this cross-sectional study at two tertiary hospitals, Freetown, Sierra Leone, we observed hand hygiene compliance (defined as using handwash with soap and water or alcohol-based hand rub (ABHR) amongst healthcare workers between June and August 2021. Using the WHO Hand Hygiene tool, observations were made in relation to the type of opportunity, different wards and types of healthcare worker. Overall, 10,461 opportunities for hand hygiene were observed, of which 5086 (49%) resulted in hand hygiene actions. ABHR was used more often than handwash (26% versus 23%, *p* < 0.001). Overall, compliance was significantly better: after being with a patient/doing a procedure than before (78% after body fluid exposure risk compared with 24% before touching a patient—*p* < 0.001); in Paediatric (61%) compared with Medical wards (46%)—*p* < 0.001; and amongst nurses (52%) compared with doctors (44%)—*p* < 0.001. Similar patterns of compliance were observed within each hospital. In summary, hand hygiene compliance was sub-optimal, especially before being with a patient or before clean/aseptic procedures. Improvement is needed through locally adapted training, hand hygiene reminders in wards and outpatient departments, uninterrupted provision of ABHR and innovative ways to change behaviour.

## 1. Introduction

Healthcare-associated infections are a major threat to patient safety and are associated with prolonged hospital stays, long-term disability, increased resistance of microorganisms to antimicrobials, poor clinical outcomes, large additional costs to health systems and unnecessary deaths [1]. The limited available evidence suggests that the burden of healthcare-associated infections is much higher in low and middle-income countries than in high-income countries due to several issues that include insufficient financial resources, scarcity of training, limited microbiological services and other competing healthcare priorities [2].

The hands of healthcare workers play a pivotal role in the transmission of microorganisms responsible for healthcare-associated infections [3], and therefore, global efforts to reduce the burden of these infections have focused on hand hygiene. These include the World Health Organization (WHO)’s campaigns “clean care is safe care” and “fight antibiotic resistance, it’s in your hands” [4,5]. These campaigns are mainly based on improving hand hygiene practices in health care settings through the implementation of the WHO multimodal hand hygiene strategy [6]. Over the last two decades, an increasing body of evidence has accumulated to suggest that improved hand hygiene can reduce healthcare-associated infections [3,7].

Hand hygiene practices can be measured using a WHO standard observation tool [8], which defines five opportunities or “moments” for hand hygiene within the health care setting. These include hand hygiene procedures (i) before touching a patient, (ii) before the performance of clean/aseptic procedures, (iii) after bodily fluids exposure risk, (iv) after touching a patient and (v) after touching a patient’s surroundings. Recent studies in Rwanda, Ghana, Nigeria and neighbouring Guinea using the WHO hand hygiene observation tool have shown low levels of hand hygiene compliance, which can be improved after training and education, provision of alcohol-based hand rub (ABHR) at points of care and placing hand hygiene reminders in the workplace [9,10,11,12].

Sierra Leone has a National Strategic Plan to Combat Antimicrobial Resistance (2018–2022) [13], which clearly outlines infection prevention control (IPC) activities and provides guidance about quarterly supportive visits to health facilities to ensure minimum IPC standards, including hand hygiene. In resource-limited settings, there are important challenges to good hand hygiene compliance that include lack of infrastructure (e.g., functioning sinks), overcrowding in hospital wards, shortages of ward basins with water and soap or ABHR, different hand hygiene techniques and cultural factors [14]. Since 2015, the National IPC Unit, supported by its partners, has provided handwash items (including soap, veronica buckets (buckets of water with a tap fixed at the bottom, mounted at hand height and with a bowl at the bottom to collect waste water) and paper towels) to various institutions, including health facilities. A boost to this hand hygiene initiative occurred in 2019 with the local production of ABHR supported by partners, and this has since been widely distributed in the country.

At the time of conducting this study, there was no published study about compliance with hand hygiene practices in the country. The aim of the study, therefore, was to assess hand hygiene compliance amongst different categories of health care workers and different wards/departments at two tertiary hospitals (34 Military (34 MH) and Connaught) in Freetown, Sierra Leone, over a three-month period between June and August 2021. The specific objectives were to assess and document hand hygiene compliance (defined as using either handwash with soap and water or ABHR) amongst health care workers. This included: (i) the overall level of compliance stratified by the two hospitals, and (ii) within each hospital, levels of compliance in relation to the five opportunities for hand hygiene action as outlined in the WHO tool, the different wards and type of health care worker.

## 2. Materials and Methods

### 2.1. Study Design

This was a cross-sectional study using primary data obtained with a standardised WHO observation tool [9].

### 2.2. Setting

#### 2.2.1. General Setting 

Sierra Leone is a country on the southwest coast of West Africa bordered by Liberia to the southeast and Guinea to the northeast. The country has a tropical climate, with a diverse environment ranging from savanna to rainforests. Sierra Leone occupies an area of about 72,000 square kilometres with a total population of just over 7 million [15]. The country is divided into five administrative regions, which are further subdivided into sixteen districts. Sierra Leone has a three-tier health care system of primary, secondary and tertiary health care. The overall responsibility of managing and organising health care services is under the Ministry of Health and Sanitation. Health care services are largely provided by the public sector, with some services provided by the private sector, non-governmental organisations, faith-based organisations and the military sector.

#### 2.2.2. Site-Specific Setting

The study sites were 34 MH and Connaught Hospital, both of which are tertiary care hospitals situated in Freetown. Site 34 MH has 200 beds, which cater for military personnel and their dependents, as well as the general civilian population. The 34 MH admits about 2000 patients per year [16]. Connaught Hospital has 300 beds spread between different wards, caters largely for civilians and admits about 4900 patients per year [17]. Both hospitals are challenged with inadequate infrastructure and lack of access to clean running water. Veronica buckets have been brought in to help solve the problem, although refilling these can be challenging.

The hospitals each have an IPC focal person appointed by the National Infection Prevention and Control Unit (NIPCU). With support from hospital management, the focal persons further identify IPC link personnel (mostly nurses, around 12 in each hospital) in each ward to support the implementation and audit of IPC practices. One of the responsibilities of these personnel is to conduct quarterly audits of hand hygiene compliance amongst health care workers, although this is infrequently done in the routine setting.

### 2.3. Study Population

The study population included healthcare workers based at two tertiary hospitals (34 MH and Connaught), Freetown, Sierra Leone, in whom hand hygiene practices were observed between June and August 2021. We intended to record all the hand hygiene opportunities that were observed during the sessions by the IPC link personnel between June and August (we estimated there would be about 10,000 opportunities over this time). Based on an estimated hand hygiene compliance of 50% [10,11,12,13] and a 95% confidence interval, the margin of error for our estimate of total hand hygiene compliance would be ±1%.

### 2.4. Study Procedure

In both hospitals, the IPC link personnel, supported by the hospital IPC focal person, observed hand hygiene compliance in the Accident and Emergency departments, the Medical Wards, the Surgical wards, Paediatric wards and Obstetrics and Gynaecology wards. Hand hygiene compliance was also assessed in the Intensive Care Unit at Connaught Hospital (no such unit existed at 34 MH). The healthcare workers observed were doctors, nurses, nursing students and laboratory technicians as they performed routine patient care.

### 2.5. Measuring Hand Hygiene Compliance

There were 24 IPC link personnel in both the hospitals, supported by the hospital IPC focal person. Half of them were involved in the first 45 days of the study (around 30 working days) and the remaining half in the next 45 days. Assuming each IPC link person records at least one session per working day (during their routine work shifts), we expected around 720 sessions (30 work days × 12 link personnel × 2 halves).

Prior to the data collection that started in June 2021, refresher training on the use of the WHO Hand Hygiene Observation Tool was conducted for the IPC link personnel who were involved in the collection of data during the observation sessions. They then used the paper-based standard observation tool in the WHO Hand Hygiene Technical Reference Manual to measure hand hygiene compliance [8]. During observation sessions, each of which took place over 10–15 min, the IPC link person quietly observed and recorded hand hygiene practices in the paper-based tool without the health care worker being made aware of this observation. This was to prevent or reduce inherent limitations and biases (such as the Hawthorne effect, where people change their behaviour because they know they are being observed) [18]. Personal information (name, ward and contact number) of the link nurses completing the hand hygiene observation tool were collected so that in the event of not being able to read the data entries, they could be contacted.

### 2.6. Data Variables

In brief, a record was made of (i) hospital name; (ii) the professional category of healthcare worker being observed (doctors, nurses, nursing students, laboratory technicians); (iii) the type of ward in which the observation was made; (iv) the opportunity that motivated the hand hygiene action (before touching a patient (bef-pat); before a clean/aseptic procedure (bef-asept); after body fluid exposure risk (aft-b.f.); after touching a patient (aft-pat); and after touching a patient’s surroundings (aft.p.surr.)); and (v) the hand hygiene action—ABHR, handwashing with soap and clean water, no hand hygiene action (classified as missed).

### 2.7. Analysis and Statistics

Data were single-entered and analysed using EpiData (version 3.1 for entry and version 2.2.2.183 for analysis, EpiData Association, Odense, Denmark). Hand hygiene compliance was calculated as a percentage: handwash actions as the numerator and opportunities for hand hygiene actions as the denominator. Similar compliance calculations were performed for the categories of handwash and ABHR and for the five opportunities that applied. Overall hand hygiene compliance was compared between the two hospitals. Hand hygiene compliance was also compared within each hospital in relation to the five opportunities for hand hygiene actions (before touching a patient was the selected referent), the different hospital wards (the medical ward was the selected referent) and the different types of health care worker (doctor was the selected referent). All comparisons (between and within the two hospitals) were assessed statistically using the chi-square test, with levels of significance being set at 5% (*p* < 0.05, two-tail).

## 3. Results

A total of 10,461 opportunities for hand hygiene actions were observed over 423 sessions (an average of 25 opportunities per session). Of these 423 sessions, 84 (20%) took place in 34 MH and 339 (80%) in Connaught hospital. Of 10,461 hand hygiene opportunities, 2072 (20%) were from 34 MH and 8389 (80%) from Connaught hospital.

### 3.1. Hand Hygiene Actions: Overall and Stratified by the Two Hospitals

Overall, hand hygiene actions were carried out in 5086 (48.6%, 95% confidence interval: 47.6, 49.6) of the 10,461 opportunities. Of 2072 opportunities observed in 34 MH, 838 (40%) resulted in hand hygiene actions: this was significantly lower than the 4248 (51%) hand hygiene actions observed in the 8389 opportunities in Connaught hospital (*p* < 0.001).

Overall, ABHR was used more often than handwash (26% versus 23%, *p* < 0.001). There was less use of both handwash (20% versus 23%, *p* < 0.01) and ABHR (20% versus 27%, *p* < 0.001) in 34 MH when compared with Connaught hospital.

### 3.2. Hand Hygiene Actions in Relation to Five Opportunities

Hand hygiene actions in relation to the five opportunities and in relation to handwash or use of ABHR are shown in Table 1. Overall, the lowest hand hygiene compliance was before touching a patient (24%), followed by before a clean/aseptic procedure (34%). In comparison, hand hygiene compliance was significantly better after the risk of body fluid exposure (78%), after touching a patient (65%) or after touching a patient’s surroundings (57%)—*p* < 0.001.

Similar differences were observed with respect to hand hygiene actions, the use of hand wash and the use of ABHR within each of the two hospitals.

### 3.3. Hand Hygiene Actions in Relation to Hospital Wards

Hand hygiene actions in relation to hospital wards are shown in Table 2. The highest level of hand hygiene compliance was observed in the Paediatric ward, overall (61%) and within each hospital (34 Military, 54%, and Connaught, 63%). Conversely, the lowest level of hand compliance was observed in the Obstetrics/Gynaecology ward, overall (34%) and within each hospital (34 MH, 34%, and Connaught, 36%). Using the Medical ward as referent, hand hygiene compliance for handwash and ABHR was significantly better overall and within each hospital in the Paediatric ward. Hand hygiene compliance for handwash and ABHR was also generally low in the Obstetrics/Gynaecology ward, especially for handwash. Of note, in 20% of opportunities for hand hygiene actions, the ward was not recorded.

### 3.4. Hand Hygiene Actions in Relation to Type of Health Care Worker

Hand hygiene actions in relation to types of health care worker are shown in Table 3. Overall, nurses had the best hand hygiene compliance (52%). This was also found in Connaught Hospital (56%), although in 34 MH, the nursing students had the best performance (48%). Laboratory technicians had the worst hand hygiene compliance overall (20%), and this was also observed in each of the two hospitals (34 MH, 16% and Connaught, 21%). With respect to handwash, nurses had the best compliance overall (28%), and this was found in each of the two hospitals. However, with respect to ABHR, doctors had the best compliance overall (33%), and this was also observed in Connaught hospital. Laboratory technicians had the worst hand hygiene compliance, whether with handwash or ABHR.

## 4. Discussion

This observational study in two tertiary hospitals in Freetown, Sierra Leone, identified three key findings with respect to hand hygiene compliance.

First, we observed compliance to hand hygiene actions in nearly half of the 10,000 or more opportunities that presented themselves. Just after this study was started, another assessment of hand hygiene compliance was conducted in two secondary hospitals in the country, one in Freetown and one in the northern region [19]. In these two hospitals, despite the reasonably constant supply of soap and ABHR, hand hygiene compliance was found to be inferior at 19% overall. Hand hygiene compliance in our two tertiary hospitals was much higher than in several recent reports from tertiary care facilities in Nigeria, Ethiopia and Kenya, where compliance levels ranged from 17% to 31% [20,21,22,23,24]. Our findings, however, were very similar to observations made in a University Teaching Hospital complex in southwest Nigeria and in over 100 health facilities in Ghana, where compliance averaged at about 50% [25,26]. All these studies used the same WHO tool. In our study, ABHR was used more frequently than hand wash with soap and water. We do not know the precise reasons for this, but it may be due to better availability of ABHR, easier use of ABHR and a perception that ABHR is more effective than simple handwashing. In one Nigerian facility, ABHR was the predominant practice for hand hygiene [11], while in another facility, it was used very occasionally in contrast to hand wash [21].

Second, hand hygiene compliance, whether with handwash or ABHR, was much higher after contact or exposure to patients, their surroundings or body fluids compared with before patient contact and before aseptic procedures. These findings align completely with previous reports from other African countries [11,12,21,22,23,26], as well as the report from the two secondary hospitals in Sierra Leone [19]. It is well recognised in these examples that health care workers are more likely to perform hand hygiene actions with self-protected opportunities (namely, after touching patients or after body fluid exposure) than with patient protective opportunities (namely, before touching a patient or before an aseptic technique). However, another explanation in busy wards may be that once a health care worker has washed his/her hands after touching a patient, he/she may feel there is no need to repeat the procedure before seeing and touching the next patient. Whatever these reasons might be, there is a need for education and training, as well as for reminders and feedback on hand hygiene practices in the workplace, and there also needs to be more focus on patient-protective hand hygiene opportunities. These interventions would be more effective and useful if there was a better understanding of the reasons and motivations for handwashing in the country. Further systematic research in this area is strongly encouraged.

Third, hand hygiene compliance differed between the workplace within the hospital and between different cadres of health care worker. In our study, hand hygiene compliance in both hospitals was best in the Paediatric wards and worst in the Obstetrics and Gynaecology wards. This was different in the other Sierra Leone study where the hand hygiene compliance level in the Paediatric wards was inferior to many other wards [19]. In other countries, little difference was found between departments and wards [21,23]. In our study, nurses and nursing students performed better than doctors, with laboratory technicians being the worst. These findings are similar to those reported elsewhere [21,23], although in the other Sierra Leone study, in one hospital, doctors outperformed nurses, while in the other hospital, the opposite was found [19]. Although hand hygiene products had been widely distributed within each hospital prior to the study, we did not investigate their availability or the support structures, such as hand hygiene posters or health care worker leadership in IPC, all of which may have influenced the take-up of hand hygiene practices. It is a well-known phenomenon that medical doctors are less likely to practice hand hygiene compared with other cadres of staff [27,28]. It is unclear why laboratory technicians performed badly in our study, and this requires further investigation.

The strengths of our study were the large number of hand hygiene opportunities compared with the previous studies and the conduct and reporting of our study according to the STROBE (Strengthening the Reporting of Observational Studies in Epidemiology) guidelines statement [29].

There were, however, some limitations. First, we had expected to have about 720 observations sessions, which, at an average of 13–15 opportunities per session, would have given us about 10,000 hand hygiene opportunities to observe. In the event, we had only 420 sessions, but this was made up with more than the expected number of opportunities per session (25 per session), giving us the desired sample size of 10,000. We did not document the reasons for the shortfall in the number of sessions, but it may have been due to reduced numbers of IPC link personnel due to illness from COVID-19 or necessary self-isolation. Second, with respect to the type of department/ward, this was not recorded in 20% of observations, and these missing data might have influenced our findings. Third, despite the precautions taken in silent observation, the Hawthorne effect cannot be completely ruled out [30]. Fourth, it would have been useful in both hospitals and in each ward to record the availability of hand washing stations, flowing tap water, veronica buckets, soap and ABHR, and also whether there were cloths or disposable paper towels for drying hands. Finally, our study was focused on two tertiary care hospitals and, therefore, may not be representative of what is happening in the country. At the two secondary hospitals in Sierra Leone, hand hygiene compliance was much lower than in the tertiary hospitals [19].

Despite these limitations, there are some important implications and recommendations from this study. First, in our two tertiary care hospitals in Freetown, and well as in most of sub-Saharan Africa, hand hygiene compliance is sub-optimal, especially during the patient’s protective opportunities. A recent study on bacterial antimicrobial resistance (AMR) estimated nearly 5 million deaths associated with AMR and 1.3 million deaths attributable to bacterial AMR in 2019 [31]. The highest all-age death rate attributable to AMR was in West Africa, and poor hand hygiene is one of the factors contributing to this. The provision of training, adapted to local needs, hand hygiene reminders in all wards and outpatient departments, as well as the uninterrupted provision of ABHR have been demonstrated to make a difference and significantly improve hand hygiene compliance in African health facilities [9,10,11,12]. With Sierra Leone now locally producing ABHR, the country is in an excellent position to embrace and move forward on this and improve compliance levels in all its health facilities.

Second, further research is needed to get better information on hand hygiene compliance throughout the country and at different levels of the health care system, as was done recently in Ghana [26]. We need to understand why compliance is higher for “after opportunities” than for “before opportunities” and why differences exist between wards and different health care worker cadres. This is a relatively easy and inexpensive research package to implement, which at the same time would pay dividends for Sierra Leone’s IPC activities.

Finally, the assessment of hand hygiene compliance, as well as the availability of hand hygiene facilities, should become a regular quarterly monitoring activity accompanied by supervision and oversight, with the leadership provided by the NIPCU. Innovative ways to improve hand hygiene actions, such as the use of “emojis” or “positive nudges”, constructive competition among departments, positive reinforcement and addressing a combination of determinants (social influence, attitude, self-efficacy or intention) should be considered [32,33]. These are particularly important in the current COVID-19 era, where general infection prevention and control activities in health care facilities need to be strengthened.

## 5. Conclusions

Hand hygiene compliance was assessed using the WHO hand hygiene observation tool in two tertiary care hospitals in Freetown, Sierra Leone, between June and August 2021. Out of a total of 10,461 opportunities for hand hygiene actions, compliance was found in about half, with ABHR used more frequently than hand wash. Hand hygiene compliance was significantly higher after being with a patient or doing a procedure than before. Compliance was significantly higher in the Paediatric wards and lower in the Obstetrics and Gynaecology wards, and significantly higher amongst nurses compared with doctors and laboratory technicians. In general, the patterns of hand hygiene compliance that were observed were similar in both hospitals.

## Figures and Tables

**Table 1:** Hand hygiene compliance between and within the two tertiary hospitals in relation to the opportunity (moment) for a hand hygiene action and in relation to whether this was handwash or hand rub (ABHR) in Freetown, June–August 2021.

Type of Hospital	Opportunities for Hand Hygiene Action	Hand Hygiene Actions Done		Handwash HW		Hand-Rub ABHR	
	N	n	(%)	n	(%)	n	(%)
Both hospitals							
Total opportunities:	10,461	5086	(49)	2370	(23)	2716	(26)
Bef-pat	3244	792	(24) ref	150	(4) ref	642	(20) ref
Bef-asept	1039	350	(34) **	159	(15) **	191	(18)
Aft-b.f.	1000	780	(78) **	601	(60) **	179	(18)
Aft-pat	2721	1777	(65) **	820	(30) **	957	(35) **
Aft.p.surr.	2447	1382	(57) **	638	(26) **	744	(30) **
Not recorded	10	5	(50)	2	(20)	3	(33)
34 Military Hospital							
Total opportunities:	2072	838	(40)	415	(20)	423	(20)
Bef-pat	602	120	(20) ref	51	(9) ref	69	(12) ref
Bef-asept	285	89	(31) **	43	(15) *	46	(16)
Aft-b.f.	315	237	(75) **	172	(55) **	65	(21) **
Aft-pat	473	238	(50) **	92	(20) *	146	(31) **
Aft.p.surr.	397	154	(39) **	57	(14) *	97	(24) **
Not recorded	-	-	-	-	-	-	-
Connaught Hospital							
Total opportunities:	8389	4248	(51)	1955	(23)	2293	(27)
Bef-pat	2642	672	(25) ref	99	(4) ref	573	(22) ref
Bef-asept	754	261	(35) **	116	(15) **	145	(19)
Aft-b.f.	685	543	(79) **	429	(63) **	114	(17)
Aft-pat	2248	1539	(69) **	728	(32) **	811	(36) **
Aft.p.surr.	2050	1228	(60) **	581	(28) **	647	(32) **
Not recorded	10	5	(50)	2	(20)	3	(30)

Row percentages (denominators are the values in column N); Observations made using the WHO Hand Hygiene Standard Observation Tool [8]; Bef-pat = before touching a patient; Bef-asept = before a clean/aseptic procedure; Aft-b.f. = after body fluid exposure risk; Aft-pat = after touching a patient; Aft.p.surr. = after touching a patient’s surroundings; Within each hospital, comparisons of hand hygiene actions are made using the chi-square test with Bef-pat being the referent against which the other opportunities (moments) are compared: this is for all hand hygiene actions, handwash and alcohol-based hand rub (ABHR). * *p* < 0.05; ** *p* < 0.001.

**Table 2:** Hand hygiene compliance within the two tertiary hospitals in relation to departments/wards where the hand hygiene actions were observed and whether this was handwash or hand-rub in Freetown, June–August 2021.

Type of Hospital	Opportunities for Hand Hygiene Action	Hand Hygiene Actions Done		Handwash HW		Hand-Rub ABHR	
	N	n	(%)	n	(%)	n	(%)
Both hospitals							
Total opportunities:	10,461	5086	(49)	2370	(23)	2716	(26)
Medical ward	3489	1588	(46) ref	737	(21) ref	851	(24) ref
Accident and Emergency	644	317	(49)	107	(17) *	210	(33) **
Surgical ward	2826	1409	(50) **	657	(23) *	752	(27)
Paediatric ward	341	207	(61) **	111	(33) **	96	(28)
Intensive care	536	279	(52) *	111	(21)	168	(31) **
Obstetrics/Gynaecology	499	168	(34) **	75	(15) *	93	(19) *
Not recorded	2126	1118	(53)	572	(27)	546	(26)
34 Military Hospital							
Total opportunities:	2072	838	(40)	415	(20)	423	(20)
Medical ward	401	161	(40) ref	82	(20) ref	79	(20) ref
Accident & Emergency	413	187	(45)	65	(16)	122	(30) *
Surgical ward	555	214	(39)	126	(23)	88	(16)
Paediatric ward	78	42	(54) *	20	(26)	22	(28)
Intensive care	-		-		-		-
Obstetrics/Gynaecology	468	157	(34) *	74	(16)	83	(18)
Not recorded	157	77	(49)	48	(31)	29	(19)
Connaught Hospital							
Total opportunities:	8389	4248	(51)	1955	(23)	2293	(27)
Medical ward	3088	1427	(46) ref	655	(21) ref	772	(25) ref
Accident and Emergency	231	130	(56) *	42	(18)	88	(38) **
Surgical ward	2271	1195	(53) **	531	(23)	664	(29) **
Paediatric ward	263	165	(63) **	91	(35) **	74	(28)
Intensive care	536	279	(52) *	111	(21)	168	(31) *
Obstetrics/Gynaecology	31	11	(36)	1	(3) *	10	(32)
Not recorded	1969	1041	(53)	524	(27)	517	(26)

Row percentages (denominators are the values in column N); Observations made using the WHO Hand Hygiene Standard Observation Tool [8]; Within each hospital, comparisons of hand hygiene actions are made using the chi-square test with the Medical Ward being the referent against which the other wards/departments are compared: this is for all hand hygiene actions, handwash and alcohol-based hand rub (ABHR). * *p* < 0.05; ** *p* < 0.001; Fisher Exact test used when cell numbers < 5.

**Table 3:** Hand hygiene compliance within the two tertiary hospitals in relation to the type of health care worker observed and whether this was hand wash or hand-rub in Freetown, June–August 2021.

Type of Hospital	Opportunities for Hand Hygiene Action	Hand Hygiene Action Done		Hand Wash HW		Hand-Rub ABHR	
	N	n	(%)	n	(%)	n	(%)
Both hospitals							
Total opportunities:	10,461	5086	(49)	2370	(23)	2716	(26)
Doctor	2239	973	(44) ref	242	(11) ref	731	(33) ref
Nurse	6695	3510	(52) **	1855	(28) **	1655	(25) **
Nursing students	1094	516	(47) *	241	(22) **	275	(25) **
Laboratory Technician	432	85	(20) **	30	(7) *	55	(13) **
Not recorded	2	2	(100)	2	(100)	0	(0)
**34 Military Hospital**							
Total opportunities:	2072	838	(40)	415	(20)	423	(20)
Doctor	347	131	(38) ref	31	(9) ref	100	(29) ref
Nurse	1582	664	(42)	371	(24) **	293	(19) **
Nursing students	62	30	(48)	11	(18) *	19	(31)
Laboratory Technician	81	13	(16) **	2	(3)	11	(14) *
**Connaught Hospital**							
Total opportunities:	8389	4248	(51)	1955	(23)	2293	(27)
Doctor	1892	842	(45) ref	211	(11) ref	631	(33) ref
Nurse	5112	2846	(56) **	1484	(29) **	1362	(27) **
Nursing students	1032	486	(47)	230	(22) **	256	(25) **
Laboratory Technician	351	72	(21) **	28	(8)	33	(13) **
Not recorded	2	2	(100)	2	(100)	0	(0)

Row percentages (denominators are the values in column N); Observations made using the WHO Hand Hygiene Standard Observation Tool [8]; Within each hospital, comparisons of hand hygiene actions are made using the chi-square test with the doctor being the referent against which other types of health care worker are compared: this is for all hand hygiene actions, handwash and alcohol-based hand rub (ABHR). * *p* < 0.05; ** *p* < 0.001; Fisher Exact test used when cell numbers < 5.

## Data Availability

The dataset used in this paper has been deposited at https://doi.org/10.6084/m9.figshare.19096826 (accessed on 10 February 2022) and is available under a CC BY 4.0 license.

## Disclaimer

In accordance with the WHO’s open-access publication policy for all work funded by WHO or authored/co-authored by WHO staff members, WHO retains the copyright of this publication through a Creative Commons Attribution IGO license (http://creativecommons.org/licenses/by/3.0/igo/legalcode, accessed on 10 February 2022), which permits unrestricted use, distribution and reproduction in any medium provided the original work is properly cited. There should be no suggestion that WHO endorses any specific organisation, products or services. The views expressed in this article are those of the authors and do not necessarily reflect those of their affiliated institutions. The use of the WHO logo is not permitted. This notice should be preserved along with the article’s original URL.
